# Indications for Electrophysiology Study in children

**Published:** 2008-05-01

**Authors:** Seshadri Balaji

**Affiliations:** Associate Professor, Department of Pediatrics, Division of Cardiology, Oregon Health & Science University, Portland OR USA

**Keywords:** pediatric ablation, electrophysiology, radiofrequency

## Introduction

The advent of electrophysiology (EP) testing revolutionized the care of children with arrhythmia. Precise mechanistic and anatomical diagnosis of arrhythmias became possible. The later development of catheter-based ablation transformed the care of these children by allowing many arrhythmias to be cured during the same procedure. Indications for EP testing vary depending on the age of the child, the underlying cardiac anatomy, and the suspected arrhythmia. In the current era, the indications for EPS and for ablation are virtually identical. There are a few situations where EPS is sometimes performed without the use of ablation, and these will be pointed out. This paper will address the common conditions for which EP testing is performed.

## Bradycardia

EP testing is rarely necessary to establish an exact diagnosis in bradycardic patients. Non-invasive testing such as Holter monitors, event recorders, and exercise testing are often far more helpful because they show the nature of the arrhythmia in the clinical setting.

In patients with suspected sinus node disease, if necessary the degree of sinus node dysfunction can be categorized by testing the response of the sinus node to overdrive suppression (the so-called sinus node recovery time). However, it is rare that the same patient does not already have demonstrable sinus bradycardia or pauses on a 24 hour ambulatory ECG (Holter). Sometimes it can be hard to decide whether sinus bradycardia is secondary to a high vagal tone or to an intrinsically abnormal sinus node. In such cases, determination of the sinus node recovery time with and without autonomic blockade can help differntiate the two conditions. In practice, the need for this is extremely rare.

AV node dysfunction can be divided into supra or infra Hissian block. Supra-Hissian block shows up on ECG monitoring as Wenckebach while infra-Hissian block shows up as Mobitz type 2 block. In the latter group, abnormalities of conduction in either bundle branch are often present. In rare patients, His bundle recordings can help determine the exact site of block. This can have prognostic implications since infra-Hissian block more often progresses to complete block and needs to be treated more aggressively. However, it is rare that EP testing is necessary in order to determine the site of block or the need for pacing.

## Tachycardia

### Supra-ventricular tachycardia

Supra-ventricular tachycardia (SVT) is the commonest indication for EPS in children. It is the commonest arrhythmia and, with the advent of catheter ablation, is often successfully cured during the combined EP/ablation procedure.

Since its initial advent in 1990, catheter ablation results have improved significantly. Currently, success rates for simple arrhythmias such as AVNRT is close to 100%, with a very low complication rate [1]. This makes it possible to justify the use of EP procedures in almost anyone with diagnosed SVT. However, certain caveats still apply.

The risk of complications has been shown to be higher in children under the age of 4 years. Therefore, most authors [and the North American Society for Pacing and Electrophysiology (NASPE) guidelines in children] apply higher threshold for performing EP procedures in children below 4-5 years [1,2].

The indications for EP testing and for ablation are almost similar in children with SVT. Hence the NASPE guidelines for ablation can be used to also describe the indications for EPS [2]. Perhaps the only two circumstances (with respect to SVT) where EPS is performed without the intention of proceeding to ablation are: transesophageal EPS in infants who have SVT resistant to medical therapy and sudden death risk assessment in children with asymptomatic WPW.

Class I indications are those patients in whom experts in the field agree unequivocally that EP testing is beneficial and where there is evidence to prove such a position include the following: patients with incessant SVT who show signs of ventricular dysfunction, patients with WPW who have had syncope or near-miss sudden death, and patients with VT and hemodynamic compromise.

Incessant tachycardia can lead to cardiomyopathy and even death3. Therefore, patients with incessant SVT who show ventricular dysfunction are a very serious group. Catheter ablation has the potential to completely cure these patients. In children, the commonest arrhythmias that lead to cardiomyopathy are: ectopic atrial tachycardia, persistent junctional reciprocating tachycardia, and incessant ventricular tachycardia.

Incessant ectopic atrial tachycardia in infants may be a special category. There is some evidence to suggest that if the arrhythmia can be medically controlled, it could resolve spontaneously in 2 -3 years [4]. Therefore, unless the cardiomyopathy is severe, there may be some benefit in trying to achieve pharmacologic control in infants.

The risk of sudden death in WPW has been estimated to be between 1 per 1000 to 10,000 patient years [5]. More recent data suggests a higher risk but this is not based on a population-based study [6]. The cause of sudden death is thought to be the development of atrial fibrillation (there is an unexplained increase in incidence of AF in WPW patients), which is conducted to the ventricle in the presence of an accessory pathway with a short refractory period, leading to ventricular fibrillation (VF) and death. The key seems to be the refractory period of the accessory pathway being <250 ms. The implication of this is that, any patient with WPW and syncope (or near-miss sudden death) deserves and EP study with ablation [7,8].

Class IIa indications are those where experts have divergent opinions although the majority are in favor of EP testing include: medically refractory SVT in children >4 yrs, children with impending CHD surgery who have SVT, children with chronic/incessant SVT without LV dysfunction, children with chronic/frequent recurrent incisional atrial reentrant tachycardia (IART).

Class IIb indications are those where experts hold divergent opinions and there is no majority in favor of the procedure. These are: asymptomatic WPW in children > 5yrs, SVT in children > 5 yrs when medications are effective, and SVT in children <5 yrs where medications are ineffective or show side effects.

Class III indications are those where there is clear agreement that the procedure is not indicated or that risk exceeds benefit. These include: asymptomatic WPW in children < 5yrs, SVT in children < 5yrs that is controlled with conventional medications, non sustained VT with no LV dysfunction and minimally symptomatic non sustained VT, those with infrequent IART episodes, and the use of AVN ablation plus pacer in intractable IART and patients with a single episode of VT with hemodynamic compromise that is amenable to ablation.

## EP for risk stratification of sudden death in CHD patients

Risk stratification for the likelihood of sudden death (SD) in patients with CHD has remained a problem area. Since the introduction of the implantable cardioverter-defibrillator (ICD), there is now hope that SD can be prevented at least in some patients. However, accurate risk stratification is essential, since ICD therapy is not without its disadvantages. Some recent evidence suggests that EP testing can help. Khairy et al [9] reported the results of EP testing in 252 pts with tetralogy of Fallot of whom 25% had clinical VT. Monomorphic VT was inducible in 30% and polymorphic VT in 4.4%. If both forms of VT were combined for the risk stratification of sudden death, the sensitivity was 77% and specificity, 80%. The positive predictive value was 55% and the negative predictive value was 92%. While these percentages are encouraging, much work still remains, particularly in other lesions such as transposition and single ventricle.

## Trans-esophageal EPS in infants

In some infants, SVT can be hard to control even with potent medications such as the class Ic drugs (flecainide, propafenone) and the class III (amiodarone, sotalol). In such instances, trans-esophageal EPS with stimulation of the atrium to try and induce SVT can be helpful. Failure to induce SVT (while on the drug) by EPS is encouraging and can help the clinician feel more confident to allow the baby to go home. Because of the ease with which a TEEPS can be performed even at the bedside (using a TEEP catheter passed similar to a naso-gastric tube, a portable stimulator and an ECG machine), it can be repeatedly performed until a drug or a combination of drugs can be found which lead to successful non-inducibility of the SVT.

## EPS and ablation in CHD patients with SVT and VT

Numerous case series have shown the usefulness of ablation to manage SVT and VT in patients with CHD [10,11]. SVT ablations have included patients with IART in complex anatomy such as transposition of the great arteries patients with previous Mustard's or Senning's operations, or in single ventricle patients who have had the Fontan operation. The latter group is widely acknowledged to be the most complex group with the potential for the most difficult anatomy, the most complex rhythms and the thickest atriums, which can make ablation particularly difficult. Even in these patients, excellent acute success rates have been reported, although late recurrence has, so far, remained the main problem [10,11].

## Conclusions

EPS has revolutionized our understanding of arrhythmias in children. With the advent of ablation, the combination of EPS plus ablation have transformed the care of these children. EPS should be considered in every child with a symptomatic arrhythmia and even in some asymptomatic ones (such as WPW and incessant arrhythmias) since certain arrhythmias can be dangerous to the life or the well being of the child.
